# Assessment of predictive factors for acute kidney injury after thrombectomy using AngioJet for iliofemoral deep vein thrombosis

**DOI:** 10.31744/einstein_journal/2026AO2438

**Published:** 2026-08-12

**Authors:** Veruska Castanheira Frade, Vinícius Bertoldi, Manoel Augusto Lobato, Vitor Cervantes Gornati, Walter Campos, Nelson Wolosker, Igor Rafael Sincos

**Affiliations:** 1 Hospital Israelita Albert Einstein Department of Vascular Surgery São Paulo SP Brazil Department of Vascular Surgery, Hospital Israelita Albert Einstein, São Paulo, SP, Brazil.; 2 Hospital Edmundo Vasconcelos Department of Vascular Surgery São Paulo SP Brazil Department of Vascular Surgery, Hospital Edmundo Vasconcelos, São Paulo, SP, Brazil.; 3 Prime Care Medical Complex São Paulo SP Brazil Prime Care Medical Complex, São Paulo, SP, Brazil.; 4 Universidade de São Paulo Hospital das Clínicas Faculdade de Medicina São Paulo SP Brazil Hospital das Clínicas, Faculdade de Medicina, Universidade de São Paulo, São Paulo, SP, Brazil.; 5 Hospital Sírio libanês São Paulo SP Brazil Hospital Sírio libanês, São Paulo, SP, Brazil.; 6 Hospital Israelita Albert Einstein Faculdade Israelita de Ciencias da Saude Albert Einstein São Paulo SP Brazil Faculdade Israelita de Ciencias da Saude Albert Einstein, Hospital Israelita Albert Einstein, São Paulo, SP, Brazil.

**Keywords:** Acute kidney injury, Thrombectomy, Venous thrombosis

## Abstract

**Objective:**

To identify the relationship between acute kidney injury (AKI) and the treatment of iliofemoral deep vein thrombosis in the acute phase using an AngioJet® device.

**Methods:**

This retrospective study included all 40 patients diagnosed with iliofemoral deep vein thrombosis who underwent treatment with AngioJet, conducted at the Vascular and Endovascular Clinic Service (Team Science) of *Hospital Israelita Albert Einstein*(HIAE), *Hospital Edmundo Vasconcelos, Hospital São Camilo*.

**Results:**

No significance difference was observed regarding sex (p=0.128), procedures with vs. without stent placement (p=0.138), body mass index (BMI) <25 vs. >25kg/m^2^ (p=0.4), and BMI <30 vs. >30kg/m^2^ (p=0.77). Independent factors associated with the development of AKI included hemoglobin <10mg/dL in the immediate postoperative period (p=0.0009), a 2-point decrease in hemoglobin level immediately after surgery (p=0.0088), history of previous major surgery (p=0.002), and glomerular filtration rate <60mL/min/1.73m² (p=0.02) in the first postoperative day.

**Conclusion:**

A relationship between the use of AngioJet and the development of AKI was noted. Independent risk factors for AKI were hemoglobin <10mg/dL in the immediate postoperative period, a 2-point decrease in hemoglobin level immediately after surgery, history of previous major surgery, and glomerular filtration rate <60mL/min/1.73m² in the first postoperative day.

## Highlights

A relationship exists between the use of AngioJet and the development of acute kidney injury.

Independent factors associated with the development of acute kidney injury include hemoglobin level <10mg/dL in the immediate postoperative period, a 2-point decrease in hemoglobin immediately after surgery, history of previous major surgery, and glomerular filtration rate <60mL/min/ 1.73m^2^ in the first postoperative day.

## In Brief

Percutaneous pharmacomechanical thrombectomy using the AngioJet system causes hemolysis, which can be further complicated by the development of acute kidney injury. This study evaluated the frequency, risk factors, and effects of acute kidney injury in patients who underwent pharmacomechanical thrombectomy for acute deep venous thrombosis.

## Graphical abstract

**Figure:**
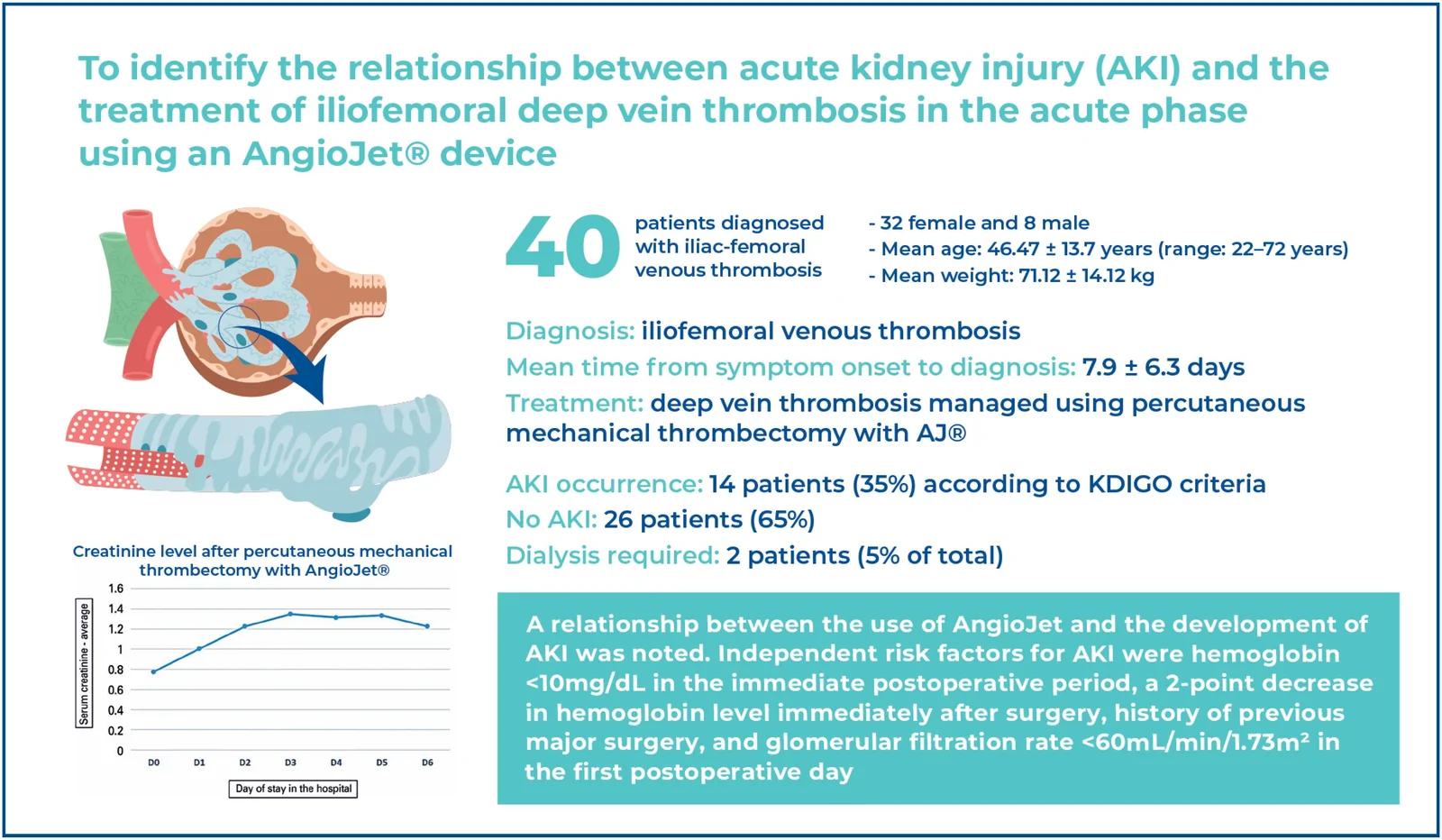

## INTRODUCTION

Deep vein thrombosis (DVT) is a common clinical disease that affects approximately 0.1% of the population worldwide. Without prompt and adequate diagnosis and treatment, it can result in serious complications.^(1)^ Early endovascular treatment for clot clearance, especially for iliofemoral DVT (IFDVT), has been shown to significantly reduce or eliminate the incidence of post-thrombotic syndrome and pulmonary embolism.^(2)^

Percutaneous mechanical thrombectomy (PMT) is an alternative treatment for DVT and includes various percutaneous devices for thrombus removal, including suction, rotation, rheolytic thrombectomy, and ultrasound. An AngioJet^®^ device is used for rheolytic thrombectomy. The potential benefits of PMT include shorter procedural time, lower thrombolytic dosage, fewer systemic effects, lower cost, and more complete resolution of the thrombus.^(3)^ Hemoglobinuria and intravascular hemolysis during PMT have garnered increasing attention from clinicians in recent years,^(4-6)^ and there is a risk of impaired renal function, which may develop into acute kidney injury (AKI).^(7,8)^

Accordingly, aggressive PMT using the AngioJet system causes hemolysis, which can be complicated further by the development of AKI.^(6)^

## OBJECTIVE

In the present study, we evaluated the frequency, risk factors, and effects of acute kidney injury in patients who underwent percutaneous mechanical thrombectomy for acute deep vein thrombosis.

## METHODS

### Sample and clinical data

Supported by our vascular team, we enrolled 40 patients who underwent treatment for acute IFDVT using AngioJet in three institutions (*Hospital São Camilo,* CAAE: 97961018.2.3002.0062, #4.170.220;*Hospital Edmundo Vasconcelos*, CAAE: 97961018.2.3001.0090, #4.187.010; and *Hospital Israelita Albert Einstein,* CAAE: 97961018.2.0000.0071, #3.315.797) between 2014 and 2018. Institutional Review Board approval was not required. Clinical data were retrieved from the hospital database system, which maintains the records of all patients, including details of medical appointments, surgical procedures, clinical course, laboratory tests, length of stay, discharge, and outpatient follow-up.

Patients were included in the study if ultrasound and digital subtraction angiography (central or mixed type) detected IFDVT and if they agreed to participate. The exclusion criteria were as follows: absolute contraindications for thrombolysis and anticoagulation, severe cardiopulmonary and renal dysfunction, allergy to contrast agents, loss to follow-up or inability to access medical records, and incomplete follow-up records.

All patients were monitored by our vascular team following the same protocols. Because the underlying disease is thromboembolic, all patients received anticoagulation with unfractionated heparin, with dosage according to their body weight. The average follow-up period was approximately 4 years.

### Definition of acute kidney injury

Regarding the definition of postoperative AKI, different studies have presented various patterns. Acute kidney injury was detected by evaluating an increase in serum creatinine concentration, rather than by a reduced glomerular filtration rate (GFR), based on the Kidney Disease Improving Global Outcomes in AKI (KDIGO AKI) clinical practice guideline.^(9)^ This guideline was created to unify the definition of AKI, based on and simplifying two previous classifications (RIFLE and AKIN),^(10)^ and records following the research protocol.

### Statistical analysis

Differences were considered statistically significant at p<0.05. The analyses were performed using SPSS for Windows, version 21.0 (IBM Corporation, Armonk, NY, USA) and GraphPad Prism (version 5.02, San Diego, CA, USA).

## RESULTS

From June 2014 to January 2018, 40 patients (32 females and 8 males; average age, 46.47±13.7 years; range, 22-72 years; weight, 71.12±14.12kg) with a diagnosis of IFDVT and an average time from symptoms to diagnosis of 7.9±6.3 days, underwent treatment for DVT using PMT via AngioJet. In this series, 14 patients (35%) developed AKI, according to the KDIGO criteria^(9)^ while 26 patients (65%) did not; only two patients required dialysis (5% of the total). One patient had a previous diagnosis of antiphospholipid syndrome, and six other patients were referred to a hematologist because of suspected thrombophilia. Ten patients had undergone previous surgery (three with thrombectomy, two with in-stent thrombosis, two with varicose vein surgery, two with Cocket syndrome correction, and one with scoliosis correction surgery).

Regarding complications from the procedure, we observed one case each of hematoma in the thigh, bleeding from the puncture site, and thrombus in the vena cava filter. Clinical improvement in the initial symptoms related to thrombosis was observed in all patients. No AngioJet-related complication or in-hospital mortality was observed (Table 1).

**Table 1:** Demographic data

		Acute kidney injury n (%)	No acute kidney injury n (%)
Sex			
	Male	1 (2.5)	7 (17.5)
	Female	13 (32.5)	19 (47.5)
Stent deployment			
	Yes	12 (30)	22 (55)
	No	2 (5)	4 (10)
BMI (kg/m^2^)			
	<25	4 (10)	12 (30)
	>25	10 (25)	14 (45)
	>30	3 (7.5)	7 (17.5)
	<30	11 (27.5)	19 (47.5)
Duration of symptoms (days)			
	<7	10 (25)	16 (40)
	>7	4 (10)	10 (25)
Age (years)			
	>60	3 (7.5)	3 (7.5)
	<60	11 (27.5)	23 (57.5)
Hemoglobin			
	<10mg/dL in POD 1	13 (32.5)	6 (15)
	>10mg/dL in POD 1	1 (2.5)	20 (50)
Hemoglobin			
	2-point decrease in POD 1	12 (30)	10 (25)
	<2-point decrease in POD 1	2 (5)	16 (40)
Multiple interventions			
	Yes	7 (17.5)	13 (32.5)
	No	7 (17.5)	13 (32.5)
Hemoglobin			
	Decrease > 15%	12 (30)	16 (40)
	Decrease <15%	2 (5)	10 (25)
Glomerular filtration rate			
	<60mg/dL in POD 1	6 (15)	3 (7.5)
	>60mg/dL in POD 1	8 (20)	23 (57.5)
Previous Surgery			
	Yes	8 (20)	2 (5)
	No	6 (15)	24 (60)
HAS			
	Yes	4 (10)	2 (5)
	No	10 (25)	24 (60)
DM			
	Yes	2 (5)	0
	No	12 (30)	26 (65)

BMI: body mass index; POD: postoperative.

Significant differences were not noted in the following factors: patient sex (p=0.164), use of stent placement (p=0.093), body mass index (BMI) <25 *versus* >25kg/m^2^ (p=0.525), BMI <30 *versus* >30kg/m^2^ (p=0.7), symptom duration >7 *versus* <7 days (p=0.533), age >60 *versus* <60 years (p=0.4), presence of multiple interventions or thrombectomies (p=1), and decrease in hemoglobin level >15% (p=0.12). Independent factors significantly associated with the development of AKI included hemoglobin <10mg/dL within postoperative day (POD) 1 (p=0.0009), a 2-point decrease in hemoglobin in POD 1 (p=0.0088), presence of previous major surgery (p=0.002), and GFR <60mL/min/1.73m² (p=0.02) in POD 1 (Table 2).

**Table 2:** Statistical analysis

	p value	95%CI
Sex	0.164	0.5249-43.699
Stent deployment	0.093	0.1460-5.7567
BMI >25kg/m^2^	0.525	0.1701-2.4699
BMI >30kg/m^2^	0,7	0.2887-6.3200
Time of symptom <7 days	0.533	0.3841-6.3562
Age > 60 years	0.409	0.3618-12.083
Multiples interventions	1	0.2727-3.6673
Hemoglobin: decrease >15%	0.1259	0.6901-20.3778
Hemoglobin <10mg/dL in POD 1	0.0009	4.6632-402.682
Hemoglobin: decrease of 2 points in POD 1	0.0088	1.7666-52.1671
Glomerular filtration rate <60mL/min/1,73 m² in POD 1	0.02	1.3268-31.032
Previous surgery	0.002	2.6734-95.7572

BMI: body mass index; POD: postoperative.

### Clinical and surgical results

After PMT with AngioJet, it was necessary to use a stent to treat residual stenosis in 34 patients (85%), including six Wallstent^®^, one Venovo^®^, seven Sioxx^®^, 12 Sinnus^®^, one Obliqus^®^, one Silver Vena, and two Ella^®^ stents. In addition to the technique, intravascular ultrasound was used in all procedures, in which a stent was placed to verify the best position. Two patients who did not properly follow the guidelines regarding the use of antiplatelet agents developed stent occlusion and were treated again.

One of the factors analyzed was the trajectory of creatinine. In our case series, we observed an increase in creatinine with increased postoperative duration. Preoperatively, the creatinine level was 0.76±0.2, and was 0.97±0.36 on POD 1, 1.16±0.66 on POD 2, 1.26±0.93 on POD 3, 1.21±1 on POD 4, 1.24±1.07 on POD 5, and 1.22±0.97 on POD 6. From POD 6 onwards, we observed a continuous decrease, as we had also observed between POD 4 and 5. In most cases, patients were discharged from the hospital after 1 week, with the exception of two patients who required hemodialysis (5%) and were hospitalized for approximately 35 days before being discharged with normal creatinine values (Figure 1). These two patients recovered full renal function with normal creatinine levels after 3 months.

**Figure 1:**
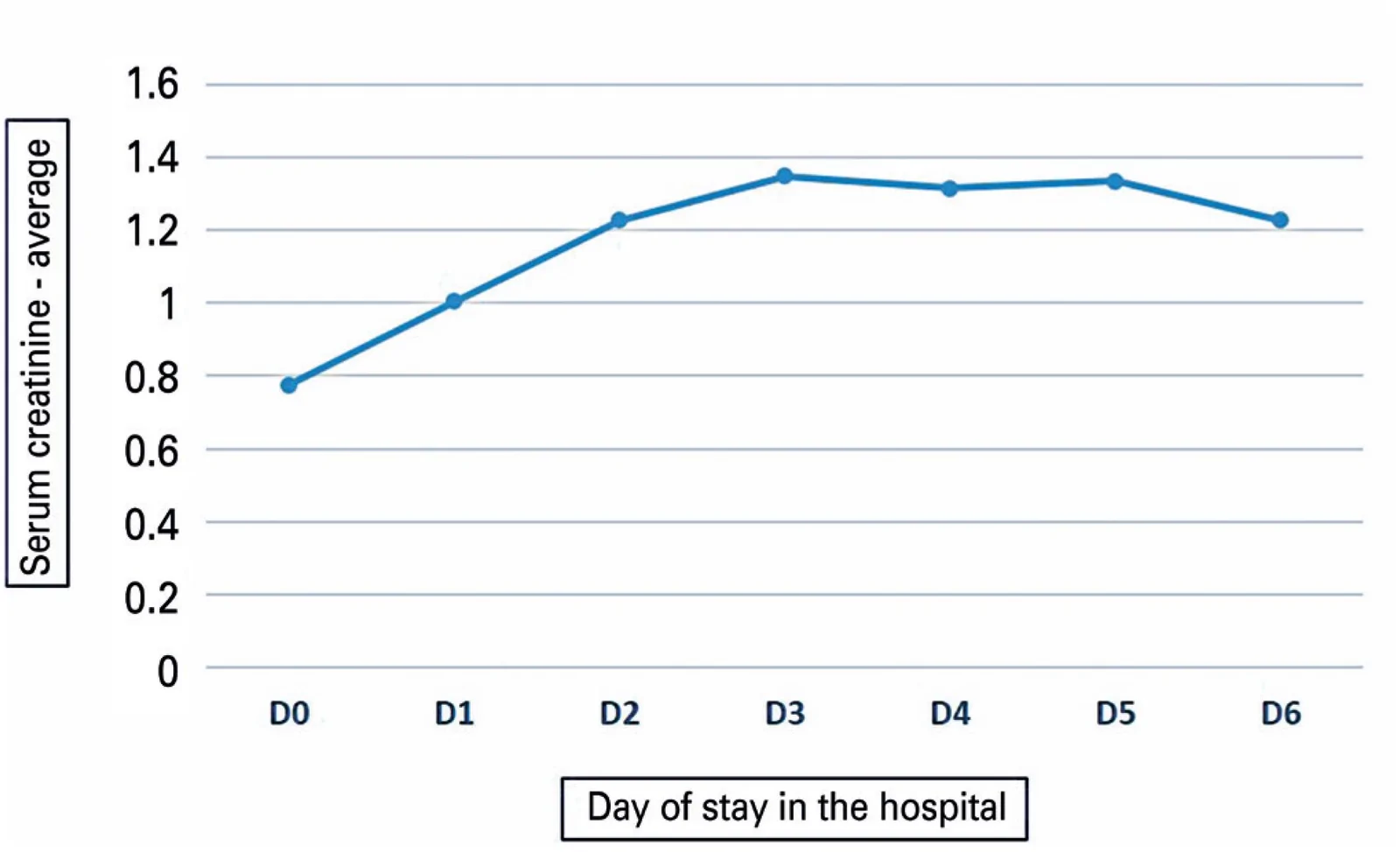
Creatinine level after percutaneous mechanical thrombectomy with AngioJet®

## DISCUSSION

Nazarian et al. demonstrated the hemolytic effect of thrombectomy in dogs and humans using rheolytic therapy.^(11)^ In humans, one of the first reports of hemolysis after the use of AngioJet was in a case of portal system thrombosis, with transient elevation in serum creatinine levels.^(12)^ The PEARL study^(13)^ briefly mentioned the association between AngioJet and the development of AKI. However, the authors did not comment on the incidence of AKI in those who did not require hemodialysis, although they stated that 5% of patients required hemodialysis 12 months post-procedure. Moreover, they did not expand on the indications for hemodialysis or the resolution and prevention of AKI in this group.^(13)^ Morrow et al. reported the incidence of AKI in patients with venous thrombosis undergoing PMT with AngioJet. They found that the incidence of renal dysfunction was significantly higher in the PMT group (21%) than in the TGC group (0%) (p=0.033). None of the patients with PMT, however, required hemodialysis.^(14)^ Similarly, Escobar et al. found that AngioJet use is an independent risk factor for AKI (odds ratio 8.22, p=0.004).^(7)^ Shen et al. also reported a significant higher risk of AKI in patients that used AngioJet for IFDVT (22.8%) than in those who underwent catheter-directed thrombolysis (9.2%) (p=0.013), respectively. Furthermore, they demonstrated that major surgery within 3 months before vascular intervention was a risk factor for AKI after AngioJet use (odds ratio 8.51, p<0.01).^(4)^

The results of the current study are similar to those already presented in the literature, with 14 patients (35%) progressing to worsening renal function. The PEARL registry's^(15)^ angular study on the topic showed that four of 283 (1.41%) patients required dialysis after AngioJet use. The current study presented similar results in terms of the hemodialysis rate (2%), and other researchers have confirmed these results.^(4)^ In contrast, another study did not report any patient progressing to dialysis, although it included fewer than 12 patients.^(16)^

A hemoglobin level <10mg/dL or a decrease in hemoglobin level below 2 points was an independent risk factor for AKI after iliofemoral thrombectomy using AngioJet. Patients with these conditions were not profoundly anemic during the treatment period, and anemia did not exceed the typical threshold for transfusion. This implies that anemia alone is unlikely to cause kidney damage. If the decrease in hemoglobin was only due to iatrogenic dilution, creatinine levels would also have decreased, reducing the probability of diagnosing AKI (in this case, we would be underestimating the incidence of AKI due to lower creatinine values). Therefore, we speculate that the greater decrease in hemoglobin in patients treated with an AngioJet may be a marker of device hemolysis, which, in turn, may contribute to the increased incidence of kidney injury in those treated with AngioJet.^(4)^ Although the exact mechanisms by which hemoglobinuria (microscopic lesion detected only in urinary sediment examination) and/or hematuria (presence of dark urine due to blood in the urine) cause kidney damage is unclear, heme-group proteins (for example, hemoglobin and myoglobin) are related to the obstruction of the renal tubules, resulting in tubular necrosis of the cells of the proximal tubule and the development of renal lesions.

Renal failure caused by acute hemolysis appears to occur following one of two scenarios. Free serum hemoglobin normally binds to plasma haptoglobin; however, when haptoglobin becomes saturated, the remaining free hemoglobin forms a dimer that is filtered in the renal tubules. Hemoglobin is reabsorbed and dissociates intracellularly into heme and globin, which are cytotoxic. The other cause of kidney damage is that heme can bind to Tamm-Horsfall proteins in the renal tubules and form molds that physically occlude tubular flow, leading to oliguria and azotemia.^(17,18)^

Major surgeries have been reported to cause postoperative AKI.^(19,20)^ By demonstrating the significant interaction between AKI and other postoperative complications in terms of costs and mortality after surgery, we aim to increase the awareness of medical staff regarding the importance of screening to identify patients undergoing surgical procedures who are at risk of postoperative AKI, as well as the need for appropriate perioperative care and postoperative management of these patients to minimize the risk of AKI.

Salem et al.^(21)^ conducted a study that included several risk factors for the development of kidney damage which were not verified in our research, such as the presence of arterial hypertension, coronary disease, and dyslipidemia. Although their research was very well conducted, with a well-defined casuistry, the factors in question cause kidney damage and can confound the analysis of independent risk factors. Therefore, in our analyses, we preferred not to consider these determinants. Interestingly, BMI showed a statistically significant difference, which we were unable to identify in our sample. As their case series had a larger sample than the current study, more studies are needed to better evaluate the factors listed above.

The use of PMT, specifically AngioJet, has brought promising results; however, as noted in our research, the risk of kidney injury is increased in these patients. We recommend that patients who present with the identified risk factors be monitored more closely, with renal function control at shorter intervals, as well as preoperative hydration. These measures would be useful as the results demonstrate.

Moreover, the medical team must be prepared for the unpredictability of an unfavorable outcome in relation to AKI, since the only preoperative parameter was a history of previous major surgery, whereas all other parameters were postoperative. One limitation of our study is that the procedure was conducted by the same team and did not involve centers from other regions. The few number of patients, despite being in agreement with several other studies, was also a limitation.

## CONCLUSION

Independent factors related to the development of kidney injury include hemoglobin <10mg/dL in the immediate postoperative period, a 2-point decrease in hemoglobin level immediately after surgery, previous major surgery, and glomerular filtration rate <60mL/min/1.73m² in the immediate postoperative period.

## Data Availability

After publication, data will be available from the authors upon request—this condition is justified in the manuscript.
